# Internet Addiction among Nursing Students and its impact on their academic performance: Case study at the Higher Institute of Nursing Professions and Health Techniques of Oujda

**DOI:** 10.1192/j.eurpsy.2025.1423

**Published:** 2025-08-26

**Authors:** S. Essadki, M. Fourtassi, O. Gouaz, N.-E. H. Sehb, R. Boughoufa

**Affiliations:** 1 Higher Institute of Nursing professions and Health Techniques, Oujda; 2 Laboratory of Life and Health Sciences, Faculty of Medicine of Tangier, Tangier, Morocco

## Abstract

**Introduction:**

Nowadays, the use of the Internet has become a very popular practice. However, excessive use can lead to serious problems such as cyberaddiction. This phenomenon has repercussions on several levels, including academic performance.

**Objectives:**

This study aimed to explore the prevalence of cyberaddiction and its associated factors among nursing students at the Higher Institute of Nursing

Professions and Health Techniques of Oujda in Morocco.

**Methods:**

A cross-sectional study was carried out among undergraduate nursing students in the city of Oujda, Morocco. The data were collected through a self-administered questionnaire that was directly distributed to all of the targeted students, exploring their demographic and academic profiles. Young scale was used to identify the levels of cyberaddiction among participants.

**Results:**

The average student’s daily connection time was 5.3+/- 3.2 hours. 26.3% of the participants were identified as cyber-dependent, and 1.4% were suffering serious issues linked to cyberaddiction. Internet addiction was significantly associated with academic failure.

**Conclusions:**

These results should lead to preventive measures in order to protect nursing students from the potential harmful effects of internet excessive use.

**Disclosure of Interest:**

None Declared

